# A patient safety knowledge graph supporting vaccine product development

**DOI:** 10.1186/s12911-023-02409-8

**Published:** 2024-01-04

**Authors:** Andrew M. Simms, Anshul Kanakia, Muhammad Sipra, Bhaskar Dutta, Noel Southall

**Affiliations:** 1 Biomedical Informatics and Medical Education, University of Washington, Seattle, Washington USA; 2 AstraZeneca PLC, Gaithersburg, Maryland USA

**Keywords:** Datasets, Knowledge graph, Pharmacovigilance

## Abstract

**Background:**

Knowledge graphs are well-suited for modeling complex, unstructured, and multi-source data and facilitating their analysis. During the COVID-19 pandemic, adverse event data were integrated into a knowledge graph to support vaccine safety surveillance and nimbly respond to urgent health authority questions. Here, we provide details of this post-marketing safety system using public data sources. In addition to challenges with varied data representations, adverse event reporting on the COVID-19 vaccines generated an unprecedented volume of data; an order of magnitude larger than adverse events for all previous vaccines. The Patient Safety Knowledge Graph (PSKG) is a robust data store to accommodate the volume of adverse event data and harmonize primary surveillance data sources.

**Methods:**

We designed a semantic model to represent key safety concepts. We built an extract-transform-load (ETL) data pipeline to parse and import primary public data sources; align key elements such as vaccine names; integrated the Medical Dictionary for Regulatory Activities (MedDRA); and applied quality metrics. PSKG is deployed in a Neo4J graph database, and made available via a web interface and Application Programming Interfaces (APIs).

**Results:**

We import and align adverse event data and vaccine exposure data from 250 countries on a weekly basis, producing a graph with 4,340,980 nodes and 30,544,475 edges as of July 1, 2022. PSKG is used for ad-hoc analyses and periodic reporting for several widely available COVID-19 vaccines. Analysis code using the knowledge graph is 80% shorter than an equivalent implementation written entirely in Python, and runs over 200 times faster.

**Conclusions:**

Organizing safety data into a concise model of nodes, properties, and edge relationships has greatly simplified analysis code by removing complex parsing and transformation algorithms from individual analyses and instead managing these centrally. The adoption of the knowledge graph transformed how the team answers key scientific and medical questions. Whereas previously an analysis would involve aggregating and transforming primary datasets from scratch to answer a specific question, the team can now iterate easily and respond as quickly as requests evolve (e.g., “Produce vaccine-X safety profile for adverse event-Y by country instead of age-range”).

## Background

From December of 2020 to the present day, billions of COVID-19 vaccine doses have been administered prophylactically to individuals around the world. Health authorities and manufacturers carefully monitored the safety of vaccines in real-time and rapidly published vast quantities of detailed data to support public health policy decisions. AstraZeneca’s Vaxzevria (ChAdOx1-S [recombinant]) vaccine received emergency use authorization in the UK on December 30, 2020, and over 2 billion doses were subsequently shipped to countries around the world in the following year [1]. Key to this ambitious, global deployment was a proactive safety monitoring approach that helped the product team anticipate safety signals as patient demographics changed from the most vulnerable at-risk populations to a universal inoculation program.

Drug safety data are organized around two primary categories, exposure and adverse events. Exposure data quantifies the population that has received the drug of interest, is typically captured at a geopolitical boundary, and can be stratified by various population demographics (e.g. age or sex). Adverse event data can be reported by anyone, including individuals receiving a drug, their families, or their health care providers [2, 3]. These reports will include the drug of interest, symptoms, and optionally other data elements such as concomitant medications and comorbidities. Exposure and adverse event data are aggregated by health authorities, and are often published as anonymized data sets. Different health authorities model their adverse event and exposure data using widely different structures, publish in different formats, and update at different frequencies. The Vaxzevria product team benefited from the active ingestion and aggregation of such health authority data sets, including both demographic profiles and spontaneous reports of adverse events for the different vaccine products. Such data provides a comprehensive, integrated view of COVID-19 vaccine safety reporting.

Adverse event and exposure data sources are summarized in Table 1. Vaccine Adverse Event Reporting System (VAERS) data contains adverse event case data for all vaccines marketed in the United States since 1990 [4, 5] and is the primary reporting tool the US Food and Drug Administration (FDA) uses for vaccine adverse event reporting. The European Medicines Agency (EMA) publishes adverse event data organized by substance the EudraVigilance system [6, 7]; however the PSKG project focused only on COVID-19 related vaccine adverse event cases since first emergency use authorizations were granted in 2020. MedDRA [8, 9] is the common terminology used in these and many other adverse event systems to codify adverse events. Exposure data for the United States is provided by the Centers for Disease Control and Prevention (CDC) through the data.gov access API [10, 11]. Exposure data for other countries is provided by European Centre for Prevention and Disease Control (ECDC) [12, 13] as well as individual health authorities.

**Table 1:** PSKG data sources formats and publication cadence

Provider	Type	Format	Access	Cadence
VAERS	adverse event	ZIP$^a$	download	weekly
CDC	exposure	table	API	daily
EudraVigilance	adverse event	Excel	Oracle gateway	daily
MedDRA	dictionary	asc$^b$	subscription download	quarterly
EMA	curated lists$^c$	Excel	download	
ECDC	exposure	Excel or XML	download	daily
Other	exposure	PDF, Excel, etc.	sent manually	varies by source

$^a$ ZIP [14] archives containing CSV [15] files, organized by year

$^b$ ASCII delimited text file format

$^c$ Designated Medical Events (DME) [16] and Important Medical Events (IME) lists [17]

Critical questions in drug safety involve the determination of whether or not a given adverse event occurs more frequently than expected in a given population. Answering these questions typically involve calculations of standardized measures such as a Proportional Reporting Ratio (PRR) [18], summary statistics, and visualizations.

We initially considered building a rich common object model to house and organize safety data, such as a data warehouse. However, these models are difficult to construct from asymmetric data sources especially when key analysis questions are not known up front. There are also complex data structures in the source data that are difficult to represent as tables. For example, the terms in the MedDRA dictionary are organized a multi-axial hierarchy, and Standard MedDRA Query (SMQ) lists are nested structures. EudraVigilance data are published with in-line updates, such that a given case may have multiple previous versions forming a linked-list structure. Further, a complex table design capable of accommodating these structures is challenging to query.

In contrast, a knowledge graph can be created and refined incrementally. It can represent tabular data easily and supports all the expected projection, transformation, and aggregation operations expected of a traditional query language. In addition, linked lists and hierarchies are represented directly, and queries can easily traverse these structures and extract information collected along pathways of interest. These features allowed us to iterate on the design and easily incorporate new data as they became needed for analysis.

## Methods

### Ontology development

We identified the primary concepts used in safety analyses and present in published data sources: cases, adverse events (as MedDRA terms), vaccines, concomitant medications, exposure (administration), and location (country and continent). Each of these concepts corresponds to a node type in the graph. Nodes were also created for the entire MedDRA term hierarchy, as well as nodes to facilitate queries grouped on arbitrary categories, such as cases reported from countries in the European Economic Association (EEA), and locally defined custom sets of MedDRA terms, (i.e., MedDRA custom queries). Finally, we added a disconnected manifest node type to enable tracking of original source files.

**Fig. 1:**
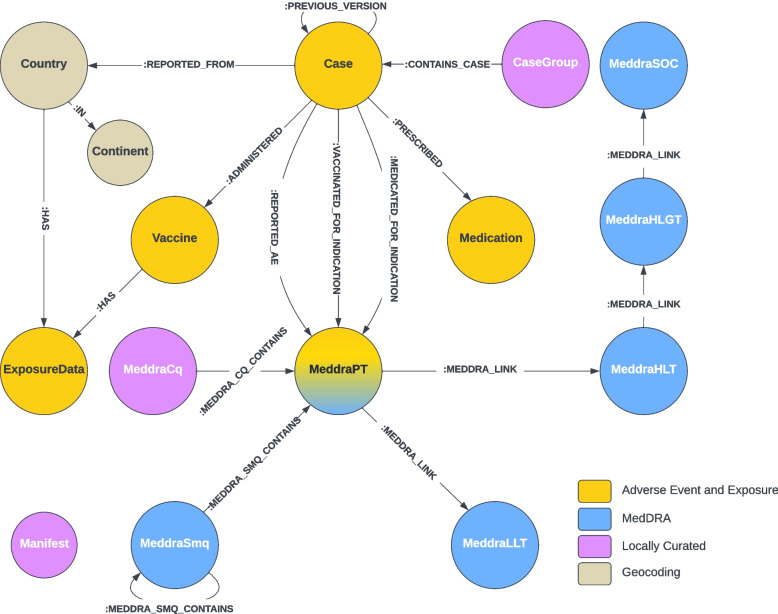
PSKG Ontology, depicting nodes categorized by data source, and relationship edges

We defined edges between concepts based on relationships present in the source data. The goal was not to replicate all the idiosyncrasies of the individual sources, but rather to identify fundamental relationships and reify these in the model as named edges. We chose reasonably descriptive but succinct names for nodes and edges in the graph, as shown in Fig. 1.

#### Cases, medications, and adverse events

A case is the fundamental unit of reporting for post-marketing surveillance. It captures one or more medications, one or more adverse events not related to the patient’s treatment, and a date. The medications and adverse events need not be causally linked. Cases may contain some demographic data about the underlying patient such as age, sex and location; additional demographic data varies by source. Although cases reported to a market authorization holder contain information that uniquely identifies a specific patient, published cases are de-identified and multiple cases may be linked to a single patient. Adverse events are coded from free text to MedDRA preferred terms, and the original text from which coded terms are derived may or may not be present in the source data.

Case demographic data, outcomes, and other categorical data are represented as tags or broken out as fields in source data. An ETL process maps categorical data to canonical values, and these values are stored as properties in the case node. Some demographic properties may be coded into a category, such as age groups in EudraVigilance cases. The ETL process will store the inclusive boundaries of the group into min_age and max_age within the case node, data sources with patient age in years as a discrete value will set both the min and max values to the given age. Patient sex is treated similarly, mapping codes or fields from source data into a unified value. The complete set of aligned case properties are shown in Table 2. A comprehensive list of node and relationship properties are listed in the Appendix in Tables 7 and 8.

**Table 2:** Additional aligned case properties

VAERS field	EudraVigilance field	Aligned property	Definition	Type
AGE_YRS	Patient Age Group	min_age	Minimum age in years	integer
AGE_YRS	Patient Age Group	max_age	Maximum age in years	integer
RECVDATE	Gateway Receipt Date	received_date	Date case received	date
SEX	Patient Sex	Gender	Patient sex	string

A case is considered serious if certain outcome criteria are met, and processing of these cases are prioritized both in reports to and cases published by health authorities. In published safety data, either the entire case or adverse events within it are labeled to indicate seriousness, with each data source defining its own indicator flags or fields. The source data fields or values used to represent seriousness are mapped to a canonical set of terms and expressed as a list at the case level as illustrated in Table 3. Some fields and tags will not align between data sources, such as the ER_VISIT (Emergency Room Visit), ER_ED_VISIT (Emergency Room or Emergency Department Visit) columns in VAERS and the “Other Medically Important Condition” tag in EudraVigilance. These are still mapped to a unified outcome value to avoid using data source specific constants in analysis code.

**Table 3:** Alignment of seriousness criteria and outcomes in source data

VAERS field	EudraVigilance tag	Unified outcome	Serious
DIED	Results in Death	death	Yes
L_THREAT	Life Threatening	life-threatening	Yes
HOSPITAL	Caused Hospitalisation	hospitalization	Yes
X_STAY	Prolonged Hospitalisation	prolonging of hospitalization	Yes
BIRTH_DEFECT	Congenital Anomaly, Birth Defect	congenital anomaly	Yes
DISABLE	Disabling, Incapacitating	disabling	Yes
ER_VISIT	-	er visit	Yes
ER_ED_VISIT	-	er visit	Yes
-	Other Medically Important Condition	other medically important condition	Yes
RECOVD	Recovering	recovered	No

**Fig. 2:**
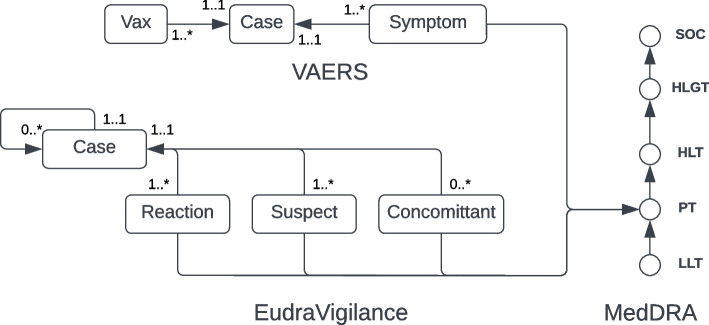
EudraVigilance and VAERS high-level native structure, and links to MedDRA

Cases are linked to other nodes through a set of edge relationships. Each case is linked to at least one preferred MedDRA term through the :REPORTED_AE edge. Adverse events in VAERS and EudraVigilance are illustrated in Fig. 2. VAERS gathers preferred terms by case in alphabetical order, a bag-of-words approach. In contrast, EudraVigilance provides a detailed list where preferred terms can be optionally accompanied by other data such as time duration. In addition, suspect and concomitant medications may also include an indication preferred term, these are recorded in the :VACCINATED_FOR_INDICATION or :PRESCRIBED_FOR_INDICATION edge. For EudraVigilance or other data sources that publish case updates, the :PREVIOUS_VERSION edge is used to link the current case to previously published versions.

Medications are captured as either vaccine or medication nodes, and are related to cases by the :ADMINISTERED or :PRESCRIBED links, respectively. Both medication node types include a brand name and generic name, an optional RxNorm concept unique identifier (RxCUI), and descriptive fields [19]. The ETL process performs rudimentary linking of drug and vaccine names from the primary adverse event data sources, VAERS and EudraVigilance; with VAERS vaccine names taking precedence. The :ADMINISTERED and :PRESCRIBED links include information date information when present in the source date, such as vaccination dates in VAERS (VAX_DATE).

#### Exposure

Exposure is a population based metric that captures the number of doses administered for a given drug. These data are typically published for a geopolitical boundary, such as a country or administrative division (province, state, county, canton) and may or may not include additional demographic stratification such as age, sex, race, ethnicity, etc. For COVID-19 vaccines requiring multiple doses, many sources provide a notion of first dose, and complete series as indicators. However, the utility of consolidated “complete” indicators were somewhat problematic as the definition of a complete dose series evolved from either one or two doses to adding boosters. Sources varied significantly in available stratification. For example, in the United States (US) the CDC provides vaccinations by vaccine type to the state and county level, and separately provides various stratification by age, sex, race for all vaccines combined. ECDC provides target group stratification, and specific categories include age ranges that vary by country as well as breaking out healthcare workers and residents of long-term care facilities. At a minimum an exposure data source is expected to provide a specific vaccine, a cumulative count of doses administered, and a country. These data are used to create ExposureData nodes and link them to the corresponding Vaccine and Country.

#### MedDRA

MedDRA is a licensed dictionary consisting of medical terms organized in a multi-axial hierarchy. MedDRA preferred terms are used in adverse event reports throughout the world by health authorities, and curated lists of MedDRA terms are used to define diseases and adverse events. Health authorities also curate terms of interest to facilitate reporting. Each MedDRA term consists of an identifier, a name, and a type. A Preferred Term (PT) is associated with one or more Low Level Terms (LLTs). Each PT is linked to one or more High Level Terms (HLTs), where one HLT is defined as the base of the primary hierarchy. HLTs link to an High Level Group Term (HLGT), and each HLGT is linked to System Organ Class (SOC) term. Figure 2 outlines this hierarchy. Standard MedDRA Queries (SMQs) are curated sets of terms. An SMQ consists of a name, a description, and links to specific preferred terms or to other SMQs. All term types and SMQs are modeled as nodes, with properties for the names and descriptions. A :MEDDRA_SMQ_CONTAINS edge is used to link SMQs to other SMQs or to specific PTs. All other links are modeled using the :MEDDRA_LINK edge. MeddraCq nodes are similar to MeddraSMQ nodes in that they store a collection of MedDRA terms. MeddraCq is used to store manually curated sets of preferred terms of interest (for reporting) that may not be present in the standard queries provided by MeddraSmq.

#### Geography, CaseGroup, Manifest

The remaining nodes in the model are Country, Continent, CaseGroup, and Manifest. Country and Continent form a simple geographic hierarchy to facilitate country based reporting. Country nodes contain basic properties such as name and International Standards Organization (ISO) abbreviations, Continents are manually curated and linked to Country node using the :IN edge. CaseGroup is used to gather case grouping information from source data, usually involving some minor transformation. For example, EudraVigilance provides a field called “Primary Source Country for Regulatory Purposes” which contains one of two fixed strings that indicate if a case originated in the EEA. This is represented in the model as an EEA or Non-EEA node, and linked to the corresponding cases. Finally Manifest nodes are used to capture information about the source data, such as the names of downloaded files and modification dates. Manifest nodes are not linked to other concepts in the graph.

### Import pipeline

**Fig. 3:**
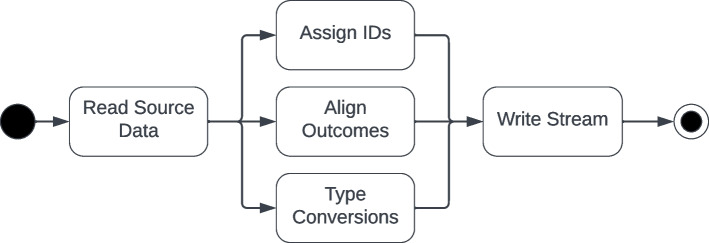
PSKG Generator class actions. Each generator reads from raw source data, assigns identifiers, align outcomes, and performs type conversions (e.g. strings to datetimes)

The import pipeline contains Python code to read data in their native format and transform them into a format suitable for importing into Neo4J [20]. Some of these data can be automatically downloaded using API calls (such as CDC exposure), others must be manually downloaded and stored locally or in Amazon Web Services, Simple Storage Service (S3) buckets. The ETL code uses a simple object model consisting of generators and pool objects. Generator objects are rooted in an abstract class that defines fundamental methods and common structures used across data sources. Pool objects manage collections of generator objects. A summary view of a generator classes for EudraVigilance and VAERS is shown in Fig. 3.

**Fig. 4:**
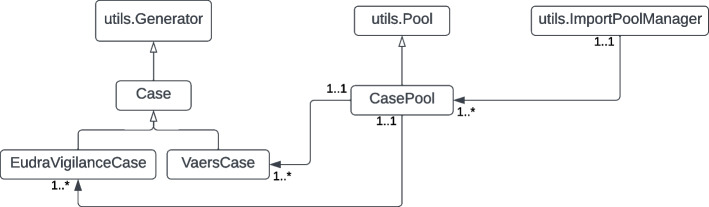
Organization of generator objects for VAERS and EudraVigilance adverse event data

Two generator instances are created per data source, one for producing nodes and one for producing edges. Both node and edge generating classes leverage shared low level utility functions that are data source specific. Generator classes are collected into pool objects. All pool instances are gathered under a pool manager. An import script creates generator instances for all source data, and registers them with the pool object for a given node or edge type. Pool objects are then registered under a pool manager object, as illustrated for cases in Fig. 4. Once all source data generator objects are created, assigned to pools, and pools registered; the script will initiate generation of load files. In the final step, all load files are made available to a Docker container hosting Neo4J. A Cypher [21] script is used to create the graph into an empty database, and perform some basic checks to mark cases that meet quality metrics. For VAERS data, only cases where the vaccination date was after December 1, 2020 and with a Time to Onset (TTO) of less than or equal to 100 days were considered for analysis. These values were chosen to eliminate cases where the patient’s birth date appeared to be entered as vaccination date, and with onset delays so long as to be unlikely related to the vaccination.

### Using the graph

The PSKG graph is accessed using Cypher queries. Cypher is a declarative pattern based query language used to extract information from a Neo4J graph database. A query consists of a series of clauses which contain Cypher statements. The initial clause is applied to the entire graph, subsequent clauses operate on the results of the previous clause. The principal statements used in clauses are MATCH and RETURN. In the simplest case, MATCH takes a pattern argument describing nodes of interest and uses a RETURN statement to specify the structure of the result set matching the pattern. A RETURN statement can directly project nodes, edges, or properties; as well as apply functions to transform results. Multiple clauses can be chained together simply by writing them sequentially. If it is necessary to calculate an interim result or helpful to only project certain values to a subsequent clause, a WITH statement can be used like RETURN to specify the precise values to pass forward. Chaining allows complex queries to be decomposed into a sequence of simple steps. The full feature set and syntax of Cypher is described elsewhere.

#### Descriptive tables and statistics

**Fig. 5:**
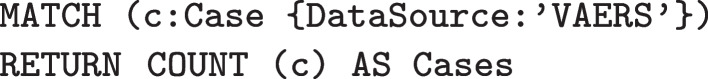
Minimal Cypher query to count all VAERS vaccine cases

Every node and edge in the graph carries a key-value dictionary for storing values, known as properties. Properties can be extracted by describing the nodes of interest using a pattern, and then specifying the desired properties in a RETURN clause. A single unqualified MATCH clause can be used to find all the nodes in a graph, and can be refined to locate specific types of nodes using a label. The match pattern can be further restricted by specifying a dictionary. Figure 5 illustrates using a MATCH clause to find Case nodes where the DataSource property is VAERS, and calculate the total number of cases.

**Fig. 6:**
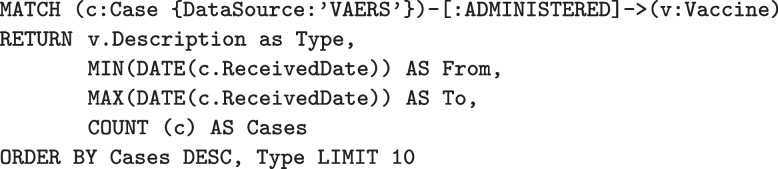
Stratifying VAERS vaccine cases by vaccine type, and returning the results ordered by descending number of cases

The simple query in Fig. 5 can be revised easily to stratify by cases by vaccine. The MATCH statement is modified to find the same Case nodes as before along with links to Vaccine nodes as shown in Fig. 6. Adding one or more property values to a RETURN clause containing an aggregate function causes the aggregation to be grouped on the non-aggregated values, producing the results shown in Table 4.

**Table 4:** The top ten highest numbers of adverse event cases reported by vaccine. The number of COVID-19 vaccination case reports eclipsed those of the second place Varicella-zoster vaccine by an order of magnitude, and is over 12 times as many reports for third place trivalent influenza vaccine, despite having been administered yearly since 1990

Vaccine	From	To	Cases
Coronavirus 2019 vaccine	12/15/2020	6/24/2022	1,320,113
Varicella-zoster vaccine	7/17/2006	6/24/2022	104,882
Influenza virus vaccine, trivalent	7/9/1990	6/23/2022	97,517
Measles, mumps and rubella virus vaccine, live	7/2/1990	6/24/2022	86,963
Varivax-varicella virus live	5/23/1995	6/24/2022	82,483
Hepatitis B virus vaccine	7/2/1990	6/23/2022	71,686
Pneumococcal vaccine, polyvalent	7/2/1990	6/24/2022	69,556
Diphtheria and tetanus toxoids and acellular p...	4/7/1992	6/23/2022	62,718
Haemophilus B conjugate vaccine	7/9/1990	6/23/2022	58,214
Human papillomavirus quadrivalent	7/14/2006	6/24/2022	46,546

#### Composition

Many safety questions involve looking at whether one adverse event is reported more frequently when another adverse event is reported. Each adverse event is typically defined as a set of MedDRA preferred terms. A given case would be included if one or more MedDRA preferred terms from the primary adverse event is present in the case. Given two adverse events, $AE_1$ and $AE_2$, each of which include multiple MedDRA preferred terms, the definition of cases with both adverse events is shown as Eq. 1. The definition of cases with $AE_1$ and without $AE_2$ is shown as Eq. 2.

1 $$\begin{aligned} C_{AE_1} \cup C_{AE_2} \end{aligned}$$

2 $$\begin{aligned} C_{AE_1} - C_{AE_2} \end{aligned}$$

**Fig. 7:**
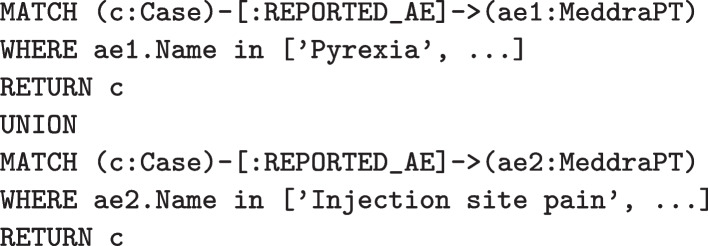
Identifying cases that include two adverse events (as defined by multiple preferred terms)

**Fig. 8:**
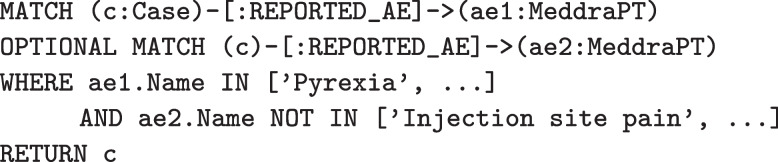
Identifying cases that include one adverse event and exclude a second adverse event (as defined by multiple preferred terms)

**Fig. 9:**
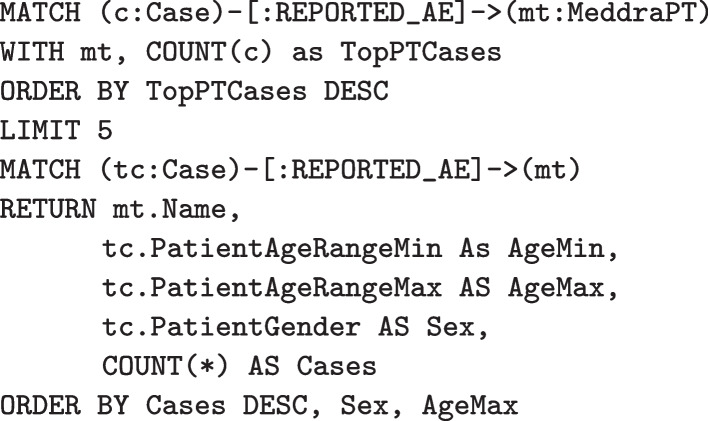
Identifying the most frequently reported adverse event terms, and then extracting case counts (stratified by age and sex). An initial MATCH statement result finds the top reported adverse event terms, and is chained to an additional clause that extracts stratified case counts

**Fig. 10:**
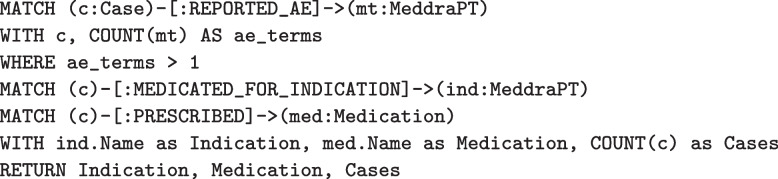
Example investigation of concomitant medications and related conditions. Here a simplistic inclusion criteria (cases with more than one adverse event term) are extracted and matched to concomitant medications and related indications, and the number of cases are summarized by indication, medication

In PSKG, these same cases can be identified using Cypher queries. Figure 7 illustrates finding all cases that include $AE_1$ or $AE_2$. Figure 8 illustrates finding all cases with $AE_1$ and without $AE_2$. As mentioned earlier, Cypher statements can produce a graph result, which can then be processed by subsequent statements using chaining. This is illustrated in Fig. 9. Here a simple query identifies the top five most frequently occurring adverse event terms, and passes this result to a second statement which then projects demographic information and counts cases involving these terms. A more complex example would be to identify a set of cases and stratify by concomitant medication and comorbidities, this is shown in Fig. 10. Here a simple filter can be used to identify cases in an inclusion set, and then count cases stratified by medications and indications.

#### Querying meta data

**Fig. 11:**
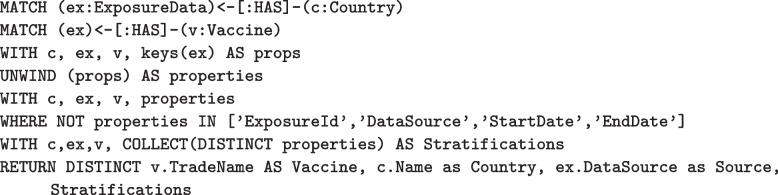
Finding available stratification for exposure data

In addition to finding data contained within the graph, the graph structure itself can be queried using Cypher. In Fig. 11, the query gathers ExposureData nodes and extracts all property keys, excluding keys not associated with stratification. A sample result from this query is shown in Table 5. The power of this query is that it will continue to function and return complete results, even if a new source of exposure data is added to the import pipeline.

**Table 5:** Limited sample output from the query in Fig. 6 illustrating available stratification by vaccine and country

Vaccine	Country	Source	Stratifications
Vaxzevria	United Kingdom	UK Govt	[GroupGender, DoseIdentifier,
			GroupAgeMin, GroupAgeMax, Count]
Spikevax	United States	CDC	[Count]
Comirnaty	United States	CDC	[Count]
Unknown	United States	CDC	[Count]
Janssen	United States	CDC	[Count]

## Results

### Graph

**Fig. 12:**
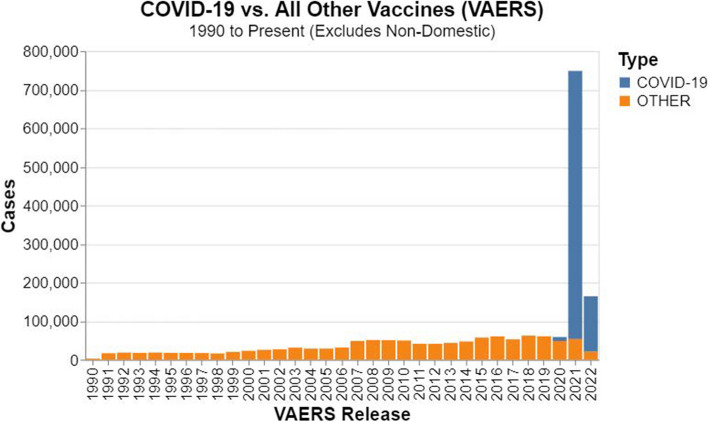
Adverse events reported in VAERS over 30 years

As of July 1, 2022 the graph contains 4,340,980 nodes and 30,544,475 edges. The breakdown of nodes and edge types are given in Table 6. Despite including over 30 years of VAERS data, the vast majority of vaccine safety information involves COVID-19 vaccines as shown in Fig. 12.

**Table 6:** Summary of PSKG Nodes and Edges as of July 1, 2022

Node	Count	Edge	Count
Case	4,209,553	PREVIOUS_VERSION	34,859
Vaccine	3,700	PRESCRIBED	1,018,016
Medication	13,848	MEDICATED_FOR_INDICATION	327,187
ExposureData	2,889	ADMINISTERED	4,709,332
Country	250	VACCINATED_FOR_INDICATION	1,602,824
Continent	6	REPORTED_FROM	4,208,850
MeddraSmq	228	CONTAINS_CASE	2,018,029
MeddraLLT	83,291	REPORTED_AE	16,470,575
MeddraPT	24,820	IN	249
MeddraHLT	1,737	HAS	5,778
MeddraHLGT	337	MEDDRA_LINK	121,606
MeddraSOC	27	MEDDRA_SMQ_CONTAINS	19,458
MeddraCq	8	MEDDRA_CQ_CONTAINS	7,712
CaseGroup	2		
Manifest	284		

### Parsing versus analysis

PSKG significantly reduced the size and complexity of analyses by refactoring parsing and cleaning code. Prior to creating the PSKG, safety scientists built custom analyses to answer specific questions. These analyses, written in Python or R, would load source data into a table structure [22, 23], perform some data cleaning, and then produce result artifact such as a table or figure. This model was adequate when source data were small, updated infrequently, and only a few analyses were needed. COVID-19 changed all these assumptions. Data sources published new data weekly or even daily, and the volume of COVID-19 vaccine eclipsed other vaccines by an order of magnitude.

#### Reduction in size

**Fig. 13:**
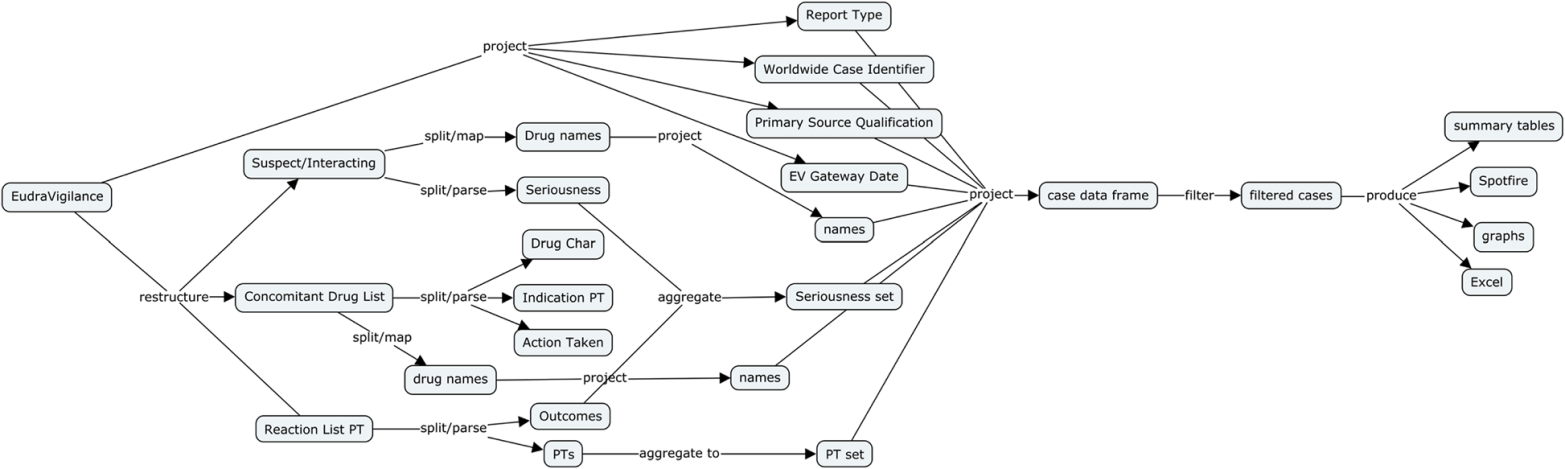
Parsing and analyzing data in EudraVigilance. EudraVigilance data are produced in a tabular form, but columns can contain complex values. Simple columns can be projected directly (e.g. Report Type), other columns such as suspect and concomitant drugs must be restructured. A typical flow is illustrated here showing the projection of simple columns to the result set, along with additional processing of multi-valued fields, some of which are combined (e.g. Outcomes and Seriousness)

A primary driver for PSKG is a complex weekly report that creates analyses based on VAERS and EudraVigilance adverse event data; and CDC and ECDC exposure data. Figure 13 illustrates the parsing and transformation processing steps involved for extracting adverse event cases into a DataFrame for EudraVigilance. The EudraVigilance export format is tabular, with one row per case. However, critical columns with suspect and concomitant drugs, as well as coded reaction terms contain multiple values per row. These complex columns must be restructured, first splitting values into lists which can be processed. Drug name variations (in this case COVID-19 vaccines) are mapped to a common name, and serious terms are split and parsed into a set. Reaction list terms are similarly split and parsed, yielding preferred terms and outcomes. Similar parsing and transformation steps are performed for each of the other data sources. When these processing steps were refactored out of the weekly analysis code and into PSKG import pipeline, the total lines of analysis code were reduced by nearly 80%. Similar reductions were seen in other analyses adapted to use PSKG, making the entire code base easier to maintain.

#### Reduction in runtime

At the onset of the pandemic, data from VAERS and EudraVigilance could be loaded, parsed and analyzed using native Python in under 20 minutes. By the summer of 2021, this time had grown to nearly 2 hours and often exceeded the capabilities of analysis laptops. Moving the loading and parsing code to the import pipeline reduced the time to produce the analyses to under 7 minutes.

#### Flexible structure

EudraVigilance, ECDC, and other exposure sources were added incrementally by aligning their data to the existing ontology structure, and extending the ontology to accommodate new relationships or concepts. In many cases extensions were made without impacting existing queries. For example, new concepts such as medication indications in EudraVigilance were added by simply defining new links (:PREVIOUS_VERSION, :VACCINATED_FOR_INDICATION, :MEDICATED_FOR_INDICATION) to the graph. The flexibility to easily accommodate new data sources while simultaneously maintaining compatibility was a significant improvement.

## Discussion

Safety questions and analyses are developed iteratively. The initial question may call for a number of cases that are associated with a set of MedDRA preferred terms. Later, those cases may need to be stratified by age, sex, or time to onset; and perhaps conditioning on different sets of preferred terms. More often than not, results from an analysis may need to be repeated at regular intervals to monitor trends.

Except in the simplest cases, analyses to answer safety questions involve the development of programs written in Python or R. These programs can be decomposed functionally into parsing, filtering, calculations, and output. In theory, the majority of the work in these programs should be the calculations. In practice, data parsing dominates all other components. The primary reasons for this are data complexity and data quality. Data sources contain complex structures including hierarchies, linked lists, and dictionaries that are encoded in vastly different formats. Data elements may be missing, contain invalid values, or deviate from published documentation.

The urgent nature of safety questions makes it tempting to quickly write programs that minimally parse a data set and transform it only enough to perform an analysis. As time goes on more and more analyses are written, parsing functions are recreated from scratch or copied from other analyses. This causes the underlying code to become very brittle-tightly coupled to a given data source and with new bugs introduced or old bugs carried along from copied sources. COVID-19 vaccine data amplified these issues, bringing unprecedented volumes of adverse event data and administration data.

**Fig. 14:**
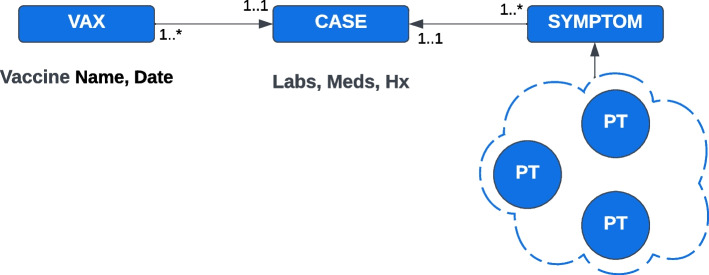
VAERS case structure illustrating how vaccination records and symptoms are linked to a case

Early in the pandemic analyses were written in Python or R to answer specific questions based on VAERS data. VAERS data is published as zip files by year, containing three comma-separated value files for case data, vaccine data, and symptom data respectively. VAERS data were originally updated quarterly, but the update frequency increased to weekly starting in late 2020. The structure of VAERS data is shown in Fig. 14, and initially was loaded in to Python as Pandas DataFrames [23, 24] with some limited transformation. Files for each year of interest can be loaded individually and then concatenated into a combined DataFrame. Pandas merge functions could then be used to query across cases, vaccines, and symptoms.

Analyses typically required iterative refining of adverse event scope to identify the most specific terms that encompassed the variety of clinical presentations associated with an event. For example, a question called for the total number of cases that showed symptoms of a Guillain-Barré Syndrome (GBS). An approach to answer this using only Pandas would be to mark cases that had any MedDRA terms associated with GBS, and a group by function with named aggregates could count up the cases. This approach becomes more challenging when investigating comorbidities, especially when conditions involve hundreds of terms.

As the question evolved, the analysis code became more complicated. For example, TTO became of interest, and analyses were parameterized to various constants such as 21 days and 14 days. Many VAERS results have invalid TTO values due to incorrect reporting dates (e.g. a birth date entered as vaccination date), these records needed to be systematically removed from consideration. Results were requested on a weekly basis, and needed to be aligned with exposure (administration) data. A separate program was developed to gather these data from the CDC data tracker website and run on a daily basis, until these data were made available using an API.

**Fig. 15:**
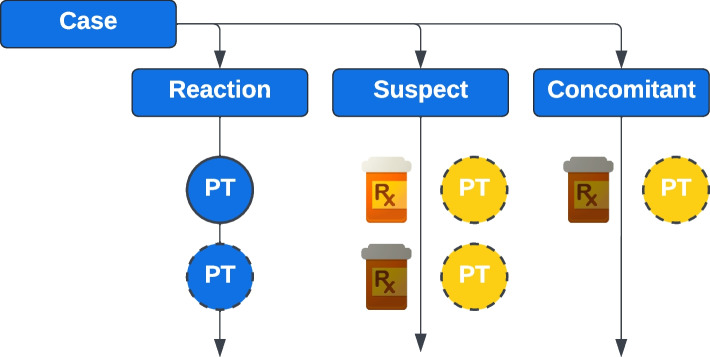
EudraVigilance case structure illustrating the reaction list preferred term(s), suspect and concomitant medication(s), and optional indication preferred terms

Analyses specific to countries in the EEA utilized data from EudraVigilance, which is produced by the EMA. EudraVigilance data also tracks adverse events, but in a very different structure that is illustrated in Fig. 15. EudraVigilance data are published in Line Listing format, which is a row oriented schema which can be saved as Comma Separated Value (CSV) or Extensible Markup Language (XML) files. However, the data cannot simply be loaded into a table as there are three multi-valued columns, and each value in these columns must be parsed using regular expressions. EudraVigilance data can contain duplicate records, which are actually updates differentiated by a receipt date. An added challenge is the system producing the line listing format is limited in the number of records it can produce in one query, and will silently truncate the results when the limit is exceeded. Thus files must be requested in chunks and reassembled in the analysis. Exposure data is produced for EEA countries by ECDC.

The first analysis was expanded to accommodate EudraVigilance and associated exposure data, but essentially had to model and parse all four different data sources into Pandas DataFrames, and then calculate various summaries and plots. Even factoring common code into packages, the code base was large and difficult to maintain. This made adding new analyses difficult, and the time spent loading and processing source data eventually grew to nearly an hour and became difficult to run on a standard analysis laptop. Although there were several avenues to consider for optimization, it was still difficult and time consuming to add new analyses or even to do exploratory work as it required intimate knowledge of each individual data source and how it was represented in DataFrames.

We considered several Business Intelligence (BI) options, but the work of assembling a relational model that could accommodate all the diverse structures found in the source data was a challenge. An On-line Analysis Processing (OLAP) dimensional style schema could be designed based on the data sets we knew about, but there are many more data sets produced by other health authorities or specific countries that would have their own unique structures. Other NoSQL solutions such as columnar stores or a Spark approach were problematic [25, 26], as they still involved mapping properties to a tabular structures not unlike DataFrames. This would result in very sparse tables or building out multi-valued columns that would need to be parsed and processed by client applications. A knowledge graph provided an elegant solution to address all of these issues, and allowed us to develop and refine the data model iteratively.

Finally, there was the question of global trends in the safety data. While individual countries’ health authorities may restrict their requests just to cases within a geo-political boundary, the overall safety profile of the product was a very important question for safety teams monitoring the vaccine rollout. Such global analysis and monitoring would be impossible without a common data model that is capable of combining and organizing data from multiple disparate data sources. This became particularly important due to the nature and speed of global vaccination programs. PSKG was instrumental for amplifying signals from multiple data sources and finding common safety trends across stratification such as age, race, sex, and concomitant medications that may otherwise have been overlooked from the individual data sources that did not share a common object model.

## Conclusions and future work

The COVID-19 pandemic prompted an unprecedented increase in the volume and frequency of public vaccine data reporting by health authorities. There is a wealth of information to be found in these data; however, this value is challenging to extract due to complex file formats and the sheer volume of data being published. In this paper we present the PSKG, a knowledge graph for organizing and analyzing vaccine safety data. We demonstrate that safety data from multiple public sources can be aligned and loaded into a graph model. Further, we show that the PSKG can both accommodate large volumes of safety data; and that analysis programs using the graph are significantly faster and easier to maintain than analysis programs bound to specific source data formats.

We intend to continue developing PSKG to accommodate additional types and sources of data, and to improve import performance. Some examples of available public data not currently loaded, and complementing existing sources in the graph, include population metrics on hospitalization, death, as well as demographics (race, age, ethnicity) of people receiving vaccinations. There are also many countries whose exposure data and vaccination data are not currently imported. Another important improvement will be the adoption of a unifying drug ontology to better align adverse event sources. We plan to expand the geocoding hierarchy, in order to more easily accommodate data stratified on political boundaries within countries such as states and counties. Many data sources such as VAERS also provide unstructured free-text fields containing case-level information such as patient comorbidities and provide additional context that could help establish causal links between adverse events and vaccines. This information is currently being investigated and a plan is being developed for using Natural Language Processing (NLP) machine learning models to extract, process, and normalize this data into the graph data model. We are particularly interested in enriching the Medication node and adding in Comorbidity nodes using this data. State-of-the-art NLP models have been trained on clinical data, such as BioBERT [27] and Clinical BERT [28, 29]. These models have been found to perform well on clinical datasets like Mimic-III [30] and can be fine-tuned for the task of extracting information from patient safety datasets.

Recent advances in graph machine learning techniques allow for tasks like link prediction on very large graphs. While the primary goal of PSKG was to support safety scientists in answering regulatory queries efficiently, the scope of this project can be expanded to pharmacovigilance. Causal and explainable link prediction algorithms could be used, not just to flag potential vaccine/adverse-event interactions but provide safety scientists with evidence within the graph for the existence of such links. Some work on adverse event prediction has been done using systems biology and mechanistic networks [31] while others have taken statistical data driven approaches using omics, social media, and electronic medical records (EMRs) [32]. More recently, the use of knowledge graphs for adverse event detection using patient health records [33], and clinical trial data [34] has gained traction. PSKG has the unique advantage of being constructed primarily using openly available data (although there are internal exposure nodes making up $<0.3$ of the total node count), thereby allowing the broader research community to develop graph machine learning algorithms and derive insights from this dataset. The increasing volume of data in both new and existing sources will eventually require significant performance improvements in the import pipeline. These improvements will most likely center around exploiting numerous opportunities for parallelism in transforming and importing source data. We hope to collaborate with the broader research community to make these and other improvements available on a regular basis. We also encourage collaboration on PSKG for developing novel explainable link prediction algorithms for post-marketing safety analysis. While considerable work has been done on graph link prediction [35], we have found that research focused on explainability of links like [36] and [37] is ripe for further advancement, particularly for healthcare applications. We aim to develop such novel models for explainable link prediction to make PSKG a truly proactive solution to pharmacovigilance and real-world drug safety monitoring.

## Data Availability

The source code and instructions for obtaining data are published on GitHub: https://github.com/AstraZeneca/PatientSafetyKG.

## Appendix

**Table 7:** PSKG nodes and properties

Node	Property	Type	Indexed	Unique
Case	CaseId	STRING	TRUE	TRUE
Case	Current	BOOLEAN	FALSE	FALSE
Case	DataSource	STRING	TRUE	FALSE
Case	DeathDate	DATE_TIME	FALSE	FALSE
Case	HospitalizationLengthInDays	INTEGER	FALSE	FALSE
Case	PatientAgeRangeMax	FLOAT	FALSE	FALSE
Case	PatientAgeRangeMin	FLOAT	FALSE	FALSE
Case	PatientGender	STRING	FALSE	FALSE
Case	PatientOutcome	LIST	FALSE	FALSE
Case	PatientRecovered	BOOLEAN	FALSE	FALSE
Case	ReceivedDate	DATE_TIME	FALSE	FALSE
Case	ReportedDate	DATE_TIME	FALSE	FALSE
Case	ReportType	STRING	FALSE	FALSE
Case	SourceCaseId	STRING	TRUE	FALSE
Case	Tag	STRING	FALSE	FALSE
Case	VaersQC	INTEGER	FALSE	FALSE
CaseGroup	Abbreviation	STRING	FALSE	FALSE
CaseGroup	CaseGroupId	STRING	FALSE	FALSE
CaseGroup	Description	STRING	FALSE	FALSE
CaseGroup	Name	STRING	FALSE	FALSE
Continent	ContinentCode	STRING	TRUE	TRUE
Continent	Name	STRING	FALSE	FALSE
Country	CountryCode	STRING	TRUE	TRUE
Country	Name	STRING	FALSE	FALSE
ExposureData	Count	INTEGER	FALSE	FALSE
ExposureData	DataSource	STRING	FALSE	FALSE
ExposureData	DoseIdentifier	STRING	FALSE	FALSE
ExposureData	EndDate	DATE_TIME	FALSE	FALSE
ExposureData	ExposureId	STRING	TRUE	TRUE
ExposureData	GroupAgeMax	FLOAT	FALSE	FALSE
ExposureData	GroupAgeMin	FLOAT	FALSE	FALSE
ExposureData	GroupGender	STRING	FALSE	FALSE
ExposureData	StartDate	DATE_TIME	FALSE	FALSE
Manifest	LastModified	DATE_TIME	FALSE	FALSE
Manifest	Path	STRING	FALSE	FALSE
Manifest	Rows	FLOAT	FALSE	FALSE
Manifest	Size	FLOAT	FALSE	FALSE
Manifest	Tag	STRING	FALSE	FALSE
MeddraCq	Abbreviation	STRING	FALSE	FALSE
MeddraCq	Authors	STRING	FALSE	FALSE
MeddraCq	CreatedDate	DATE_TIME	FALSE	FALSE
MeddraCq	Description	STRING	FALSE	FALSE
MeddraCq	Name	STRING	TRUE	TRUE
MeddraHLGT	MeddraCode	INTEGER	FALSE	FALSE
MeddraHLGT	MeddraId	STRING	TRUE	TRUE
MeddraHLGT	MeddraType	STRING	FALSE	FALSE
MeddraHLGT	MeddraVersion	STRING	FALSE	FALSE
MeddraHLGT	Name	STRING	FALSE	FALSE
MeddraHLT	MeddraCode	INTEGER	FALSE	FALSE
MeddraHLT	MeddraId	STRING	TRUE	TRUE
MeddraHLT	MeddraType	STRING	FALSE	FALSE
MeddraHLT	MeddraVersion	STRING	FALSE	FALSE
MeddraHLT	Name	STRING	FALSE	FALSE
MeddraLLT	MeddraCode	INTEGER	FALSE	FALSE
MeddraLLT	MeddraId	STRING	TRUE	TRUE
MeddraLLT	MeddraType	STRING	FALSE	FALSE
MeddraLLT	MeddraVersion	STRING	FALSE	FALSE
MeddraLLT	Name	STRING	FALSE	FALSE
MeddraPT	MeddraCode	INTEGER	FALSE	FALSE
MeddraPT	MeddraId	STRING	TRUE	TRUE
MeddraPT	MeddraType	STRING	TRUE	FALSE
MeddraPT	MeddraVersion	STRING	FALSE	FALSE
MeddraPT	Name	STRING	TRUE	FALSE
MeddraSmq	MeddraSmqCode	INTEGER	TRUE	TRUE
MeddraSmq	Name	STRING	FALSE	FALSE
MeddraSOC	MeddraCode	INTEGER	FALSE	FALSE
MeddraSOC	MeddraId	STRING	TRUE	TRUE
MeddraSOC	MeddraType	STRING	FALSE	FALSE
MeddraSOC	MeddraVersion	STRING	FALSE	FALSE
MeddraSOC	Name	STRING	FALSE	FALSE
Medication	GenericName	STRING	FALSE	FALSE
Medication	MedicationId	STRING	TRUE	TRUE
Vaccine	Description	STRING	FALSE	FALSE
Vaccine	GenericName	STRING	FALSE	FALSE
Vaccine	Manufacturer	STRING	FALSE	FALSE
Vaccine	RxNormCui	STRING	FALSE	FALSE
Vaccine	TradeName	STRING	FALSE	FALSE
Vaccine	VaccineId	STRING	TRUE	TRUE
Vaccine	VaxType	STRING	FALSE	FALSE

**Table 8:** PSKG relationships and properties

Relationship	Property	Type
ADMINISTERED	Characterization	STRING
	VaccineSite	STRING
	VaccineDate	DATE_TIME
	VaccineRoute	STRING
	Duration	FLOAT
	Dosage	STRING
	VaccineLot	STRING
CONTAINS_CASE		
HAS		
IN		
MEDDRA_CQ_CONTAINS		
MEDDRA_LINK	PrimarySoc	STRING
MEDDRA_SMQ_CONTAINS	LastModifiedVersion	STRING
	Status	STRING
	AdditionVersion	STRING
	Category	STRING
	Scope	STRING
	Weight	FLOAT
MEDICATED_FOR_INDICATION		
PRESCRIBED	Characterization	STRING
	Duration	FLOAT
	Dosage	FLOAT
	Route	STRING
	Units	STRING
PREVIOUS_VERSION		
REPORTED_AE	OnsetDate	DATE_TIME
	LengthInDays	INTEGER
REPORTED_FROM	SubRegion	STRING
VACCINATED_FOR_INDICATION		

## Abbreviations

API: Application Programming Interface
BI: Business Intelligence
CDC: Centers for Disease Control and Prevention
CSV: Comma Separated Value
FDA: US Food and Drug Administration
ECDC: European Centre for Prevention and Disease Control
EEA: European Economic Association
EMA: European Medicines Agency
DME: Designated Medical Events
IME: Important Medical Events
ETL: extract-transform-load
HLGT: High Level Group Term
HLT: High Level Term
ISO: International Standards Organization
LLT: Low Level Term
MedDRA: Medical Dictionary for Regulatory Activities
NLP: Natural Language Processing
OLAP: On-line Analysis Processing
PRR: Proportional Reporting Ratio
PSKG: Patient Safety Knowledge Graph
PT: Preferred Term
S3: Amazon Web Services, Simple Storage Service
SOC: System Organ Class
SMQ: Standard MedDRA Query
SMQs: Standard MedDRA Queries
GBS: Guillain-Barré Syndrome
TTO: Time to Onset
US: United States
VAERS: Vaccine Adverse Event Reporting System
XML: Extensible Markup Language
